# Supplementary material for: The interactome of intact mitochondria by cross-linking mass spectrometry provides evidence for coexisting respiratory supercomplexes
Source: Mol Cell Proteomics. 2017 Dec 8;17(2):216–32. doi: 10.1074/mcp.RA117.000470 (PMC5795388; doi:10.1074/mcp.RA117.000470)
Supplement: Supplemental Data [file supp_RA117.000470_133922_0_supp_23978_fzffwf.zip › spectra_annotation/mito_spectra_annotation/366-1-4-1-17-1.pdf]

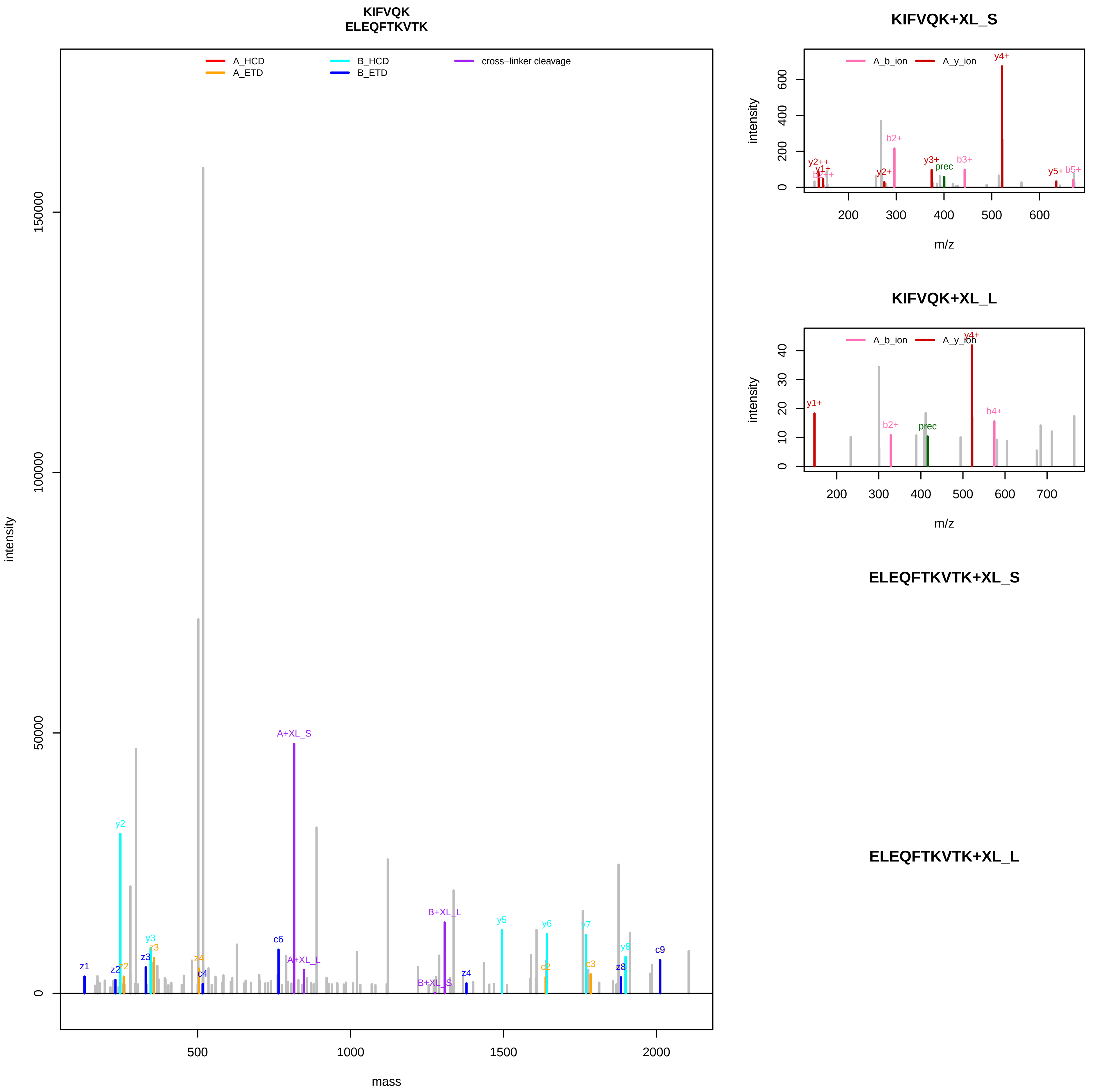
KIFVQK
ELEQFTKVTK
A_HCD
A_ETD
B_HCD
B_ETD
cross−linker cleavage
intensity
mass
0
50000
100000
150000
500
1000
1500
2000
y2
z1
z2
c2
y3
z3
c3
z4
c4
c6
A+XL_S
A+XL_L
B+XL_S
B+XL_L
z4
y5
y6
c2
y7
c3
y8
z8
c9
KIFVQK+XL_S
A_b_ion
A_y_ion
intensity
m/z
y2++
y1+
b2+
y2+
y3+
prec
b3+
y4+
y5+
b5+
KIFVQK+XL_L
A_b_ion
A_y_ion
y1+
b2+
prec
y4+
b4+
intensity
m/z
ELEQFTKVTK+XL_S
ELEQFTKVTK+XL_L
